# Management of Schneiderian Membrane Perforations during Sinus Augmentation Procedures: A Preliminary Comparison of Two Different Approaches

**DOI:** 10.3390/jcm8091491

**Published:** 2019-09-19

**Authors:** Horia Mihail Barbu, Stefania Andrada Iancu, Iasmin Jarjour Mirea, Michele Davide Mignogna, Nachum Samet, José Luis Calvo-Guirado

**Affiliations:** 1 Oral Implantology Department, Titu Maiorescu University, 031593 Bucharest, Romania; horia.barbu@gmail.com; 2 Private Clinic “Dr. Barbu Dental Clinic”, 011473 Bucharest, Romania; 3 Private Clinic “European Center of Implantology”, 011473 Bucharest, Romania; iasminjarjour@yahoo.com; 4 Head & Neck Clinical Section, Department of Neuroscience, Reproductive and Odontostomatological Sciences, Federico II University of Naples, 80138 Naples, Italy; mignogna@unina.it; 5 Department of Restorative Dentistry and Biomaterials Sciences, Harvard School of Dental Medicine, Boston, MA 02115, USA; samet@drsamet.com; 6 Department of Oral and Implant Surgery, Universidad Católica of Murcia, 30107 Murcia, Spain; jlcalvo@ucam.edu

**Keywords:** sinus floor augmentation, Schneiderian membrane perforation, sinus membrane suture, collagen membrane, accidents, long term survival

## Abstract

Background: The aim of this study was to retrospectively analyze two different sealing techniques for sinus membrane perforations produced during sinus floor augmentation by a lateral approach. Methods: A total of 172 lateral-approach sinus floor augmentation surgeries were performed on 130 patients. Sixty-one membrane perforations (35%) were reported. Most of the perforations were caused by accidental membrane tearing and 16 (26%) were caused by deliberate incision for mucocele removal. In 31 perforation cases (51%), the Schneiderian membrane was sealed by suturing, while the remaining 30 cases (49%) were sealed using a low-resorption collagen membrane coverage. Results: Out of the 31 cases treated with a suture-sealing techniques, 26 (84%) were successful, presenting graft integration. Failure occurred in the other five (16%) cases. Out of the 30 perforations sealed with low-resorption collagen membranes, 28 (93%) presented successful graft integration, while two (7%) failed. Conclusions: Both surgical techniques yielded therapeutic success.

## 1. Introduction

Since implant dentistry was firstly implemented more than 50 years ago by Professor P.I. Branemark and Professor André Schroeder, bone augmentation techniques have increasingly been indicated as creating adequate implant sites [1]. Studies have proven high survival rates for implants inserted in augmented maxillary sinuses, independent of the type of grafting material used for augmentation (autogenous bone graft, bone substitutes or a mixture of autograft and bone substitutes) [2]. However, with the increase in the number of procedures and number of doctors performing them, the incidence of intraoperative accidents and postoperative complications has also increased [3]. Schneiderian membrane perforations have been found to be the most common intraoperative complication noted in the literature [4].

A healthy and unruptured Schneiderian membrane is essential for the successful integration of bone substitute materials placed into the sinus [3]. While exposing the sinus, accidental perforations of the membrane commonly occur due to individual anatomical features, reduced membrane thickness, friability, reduced elasticity, increased adherence to the bone surface and the presence of sinus septa [5,6]. On top of that, membranes can be damaged by iatrogenic causes resulting from incorrect surgical manipulation, uncontrolled pressure on the membrane or the use of inappropriate surgical instruments [7]. Piezoelectric instruments used for antrostomy reduce the risk of perforating the Schneiderian membrane thanks to improved surgical control and the selective cutting of mineralized tissues [8,9]. Pre-surgical analysis of each individual sinus and choice of the correct surgical approach are mandatory, taking into account anatomical and personal parameters of each patient [5,10]. Lack of surgical knowledge may result in complications caused by (for instance) neglect to relate to sinus septa, which are visible on computerized tomography sections [10]. Likewise, lack of experience and reduced tactile sensitivity may increase the risk of accidental perforations. Apart from accidental perforations, there are cases in which deliberate incisions of the Schneiderian membrane are performed to remove foreign bodies or intra-sinusal entities (such as mucocele pseudocysts) that might lower the success potential of the bone-grafting procedure [11].

A successful sinus augmentation procedure requires that the bone substitute material be enclosed between a sealed Schneiderian membrane and the underlying jaw bone. Untreated Schneiderian membrane perforations result in direct communication between the added graft material and the rest of the sinus cavity, with its specific microbial flora. Thus, the compromised sterility leads to infection of the bone graft material in the maxillary sinus [7,12].

The purpose of this retrospective study was to compare two Schneiderian membrane sealing techniques that have been used in 61 lateral-approach sinus augmentation procedures.

## 2. Material and Methods

### 2.1. Study Design and Population

The study was designed as a retrospective cohort study based on intraoperative pictures and cone beam computed tomography (CBCT) images that were obtained from a digital database of a private dental clinic (the European Center of Implantology) located in Bucharest, Romania. All surgeries were performed by the same operator. Written informed consent was signed by all patients, both for treatment and for their participation in the clinical trial. The entire study followed the ethical standards of medical research that ensure and promote respect of the subjects and protect the rights and health of patients. Approval from the ethical committee (No. UTM13MAR19-MD03) was obtained by the Institutional Review Board within Titu Maiorescu University. This retrospective study followed the CONSORT guidelines.

The study included two different surgical techniques for Schneiderian membrane repair after accidental perforation or deliberate incision for mucocele removal. The surgical methods for membrane sealing were chosen based on the size and location of the perforation.

Out of this group, only patients with reported perforations of the Schneiderian membrane were included. Additional inclusion and exclusion criteria are listed in Table 1.

### 2.2. Operative Phase

The surgical phase began with local anesthesia by local infiltration, infraorbital foramen block and posterior superior alveolar nerve block using articaine hydrochloride 1:100,000 (Ubistesin Forte, 3 M ESPE, Minneapolis, MN, USA).

Following elevation of a full thickness flap, access to the sinus cavity was performed, creating an osteotomy window through the lateral wall using an ultrasound bone surgery device (Piezosurgery by Mectron s.p.a, Carasco, Italy—between 2011 and 2014, Piezomed by W&H, Bürmoos, Austria—between 2014 and 2017). Once the bony window was cut, the complete delineation of the fragment from the adjacent bony tissue was checked. The mobility of the bone fragment indicates, at this stage, that the bony window can be detached from the sinus membrane. From this moment on, perforations of the Schneiderian membrane may occur for multiple reasons such as: sharp edges of the bone window, inappropriate pressure applied with the microsaw insert, deliberate incision for mucocele removal or iatrogenic injury.

One of two sealing techniques was used to seal perforated membranes in the present study:

#### 2.2.1. Sealing technique No. I—Collagen Membrane Lining

**Figure 1:**
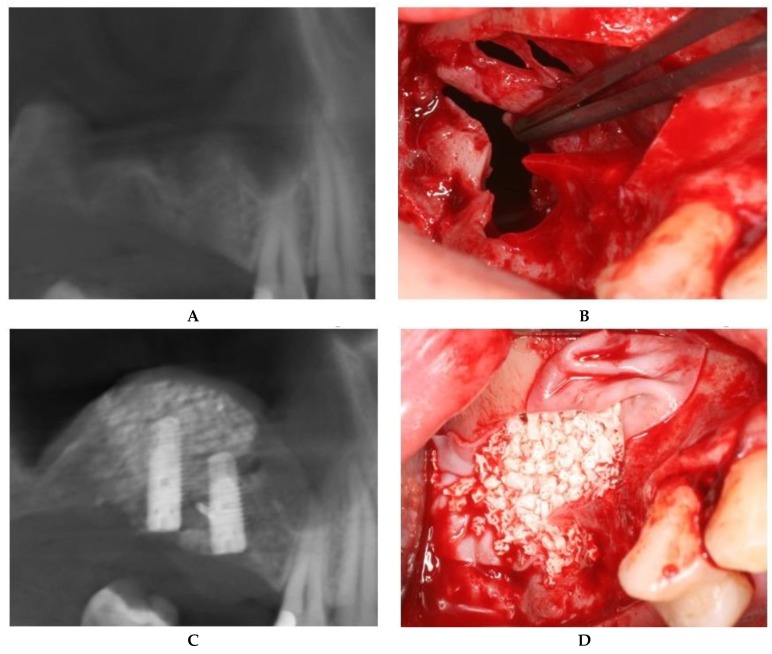
Schneiderian membrane sealing technique with collagen membrane coverage. (**A**) Preoperative CBCT image. (**B**) Large, multiple Schneiderian membrane perforations. (**C**) Bone graft placed in the collagen membrane “pouch”. (**D**) Postoperative CBCT after implant placement.

For large or multiple perforations, a single large pericardium membrane is placed in intimate contact on the elevated sinus membrane covering beyond the margin of the perforated area/s. In this study, collagen membranes with a low resorption rate (CopiOs Pericardium Membrane, Zimmer Biomet, Warsaw, IN, USA) were used to seal the perforated Schneiderian mucosa (Figure 1).

#### 2.2.2. Sealing technique No. II—Suturing Technique

**Figure 2:**
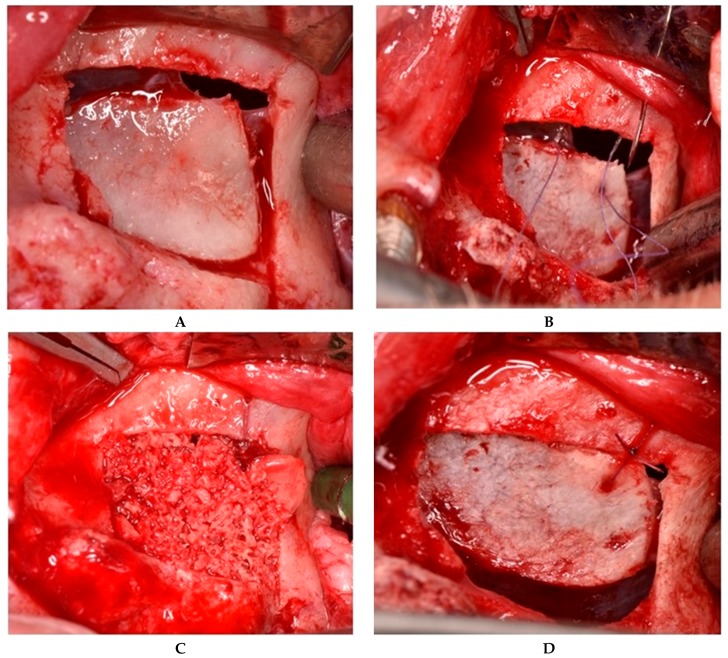
Suture of a perforation located at the superior edge of the osteotomy window. (**A**) The bone segment is still attached to the underlying mucosa. (**B**) Suturing of the Schneiderian membrane, along with the bone segment to the fixed bony plane. (**C**) “Hinge joint” of the mobile bone segment. (**D**) Grafting material introduced into the sinus cavity.

**II.a.** When sinus membrane perforation occurs at the superior edge of the osteotomy window—before detachment of the bone segment—in some cases the decision is to maintain the bone window attached to the underlying mucosa. In such cases, closure of the perforation was achieved by repositioning the osteotomy window at the upper bone edge and maintaining it in this position with a 6/0 absorbable suture and an 8-mm needle. Using a straight handpiece, holes were made with a 1-mm drill both at the level of the mobile bone segment and at a fixed point superior to the section line. An anatomical tweezer (Devemed GmbH, Tutlingen, Germany) was used to keep the mobile bone fragment in place while penetrating it with the drill. One suture with a double knot secured by two other simple knots was located on the fixed bony plane. The Schneiderian membrane was elevated from the sinus floor together with the attached bone window, with the entire structure acting as a hinge joint (Figure 2).

**II.b.** When Schneiderian membrane perforations occurred at the upper edge of the osteotomy after bone window detachment, suturing of the membrane directly to the bony edge was performed [13].

**Figure 3:**
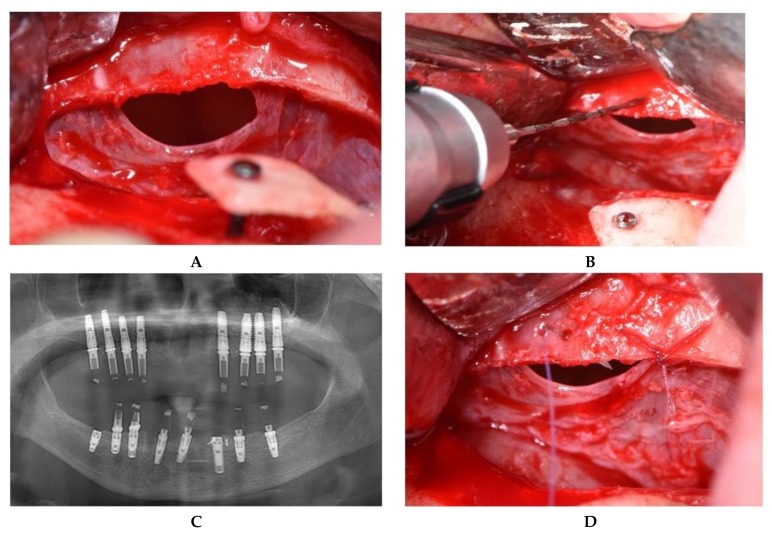
Iatrogenic perforation produced with a Langenbeck retractor. (**A**) Perforation located at the superior edge of the osteotomy. (**B**) Preparation of the holes in the bone. (**C**) Suture of the mucosa to the superior bony edge. (**D**) Postoperative panoramic image after implant placement.

Using a straight handpiece, holes were made in the cortical bone of the lateral wall at the top edge of the window level. To seal the perforation, suturing of the membrane directly to the bony edge was performed using a 6/0 absorbable thread with an 8-mm needle (Figure 3).

**Figure 4:**
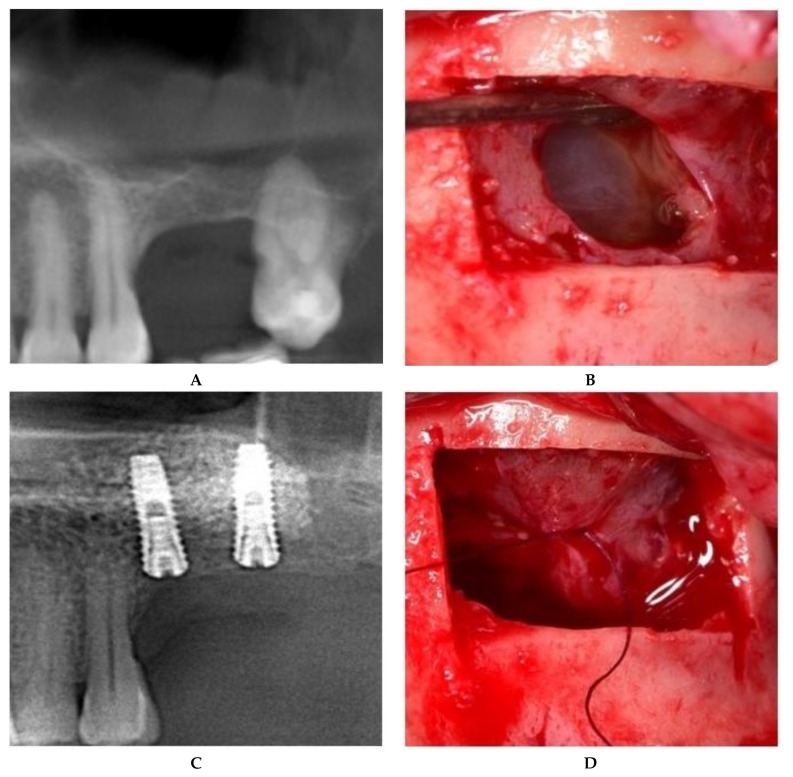
Sinus floor augmentation simultaneous with mucocele removal. (**A**) Preoperative CBCT of the left maxillary sinus. (**B**) Mucocele pseudocyst visible through the incision. (**C**) Suture of the incised Schneiderian membrane. (**D**) Panoramic image after implant placement.

**II.c.** The treatment of maxillary sinus mucoceles requires deliberate incisions of the sinus membrane. The position and size of the incision were controlled by the surgeon, both to allow mucocele removal and to further seal off the Schneiderian membrane. The surgical approach to the incised Schneiderian membrane was suturing with an absorbable 6.0 suture with an 8-mm needle (SMI Company, Belgium). Fine-tipped needle holders (Devemed GmbH, Tutlingen, Germany) and anatomical tweezers were used to create three sutures, each with one double knot secured by two other simple knots (Figure 4).

Once suturing was finished, the Schneiderian membrane was coated with a layer of PRF (Platelet Rich Fibrin), and the graft material was placed inside the sinus cavity without exerting much pressure. The grafting material used for all cases was particulate anorganic bovine bone with a 1- to 2-mm diameter (Bio-Oss, Geistlich Pharma, Wolhusen, Switzerland).

### 2.3. Postoperative Protocol

Postoperative medications included nonsteroidal anti-inflammatory drugs (dexketoprofen 25 mg) if needed, antibiotics (amoxicillin/clavulanic acid) 1 g every 12 h for 7 days and steroidal anti-inflammatory drugs (dexamethasone phosphate 8 mg) for 3 days, starting 1 day prior to surgery. Among the postoperative indications were not to sneeze with the mouth closed, not to blow the nose, and (for patients with additional bone block and bone graft material on the edentulous ridge) not to rinse with chlorhexidine. The first postoperative check was performed 48 h after the intervention, then at 7 days for suture removal and then every 25 days until the next surgical phase.

Both techniques successfully allowed bone graft integration and subsequent implant insertion 5–6 months later, achieving optimal primary stability. Four months after implant insertion, uncovering and implant loading by provisional restoration was performed. Statistical analyses were performed to compare the two sealing techniques.

## 3. Results

The sample in this study comprised 130 patients divided into 73 females (56%) and 57 males (44%) between 32 and 84 years of age, with a mean age of 52.95 years and a 10.46 standard deviation. All subjects underwent lateral-approach sinus augmentation procedures between 2011 and 2017. The patients presented no radiological and clinical signs of maxillary sinus pathologies of any kind. Patients with systemic pathologies were accepted for surgeries only if they had a good metabolic control. Out of the total of 172 sinus augmentation surgeries by the lateral approach, 61 cases (35%) presented Schneiderian membrane perforation, comprised accidental tearing (74%, corresponding to 45 patients) and intentional incision for mucocele removal (26%, corresponding to 16 patients) (Table 2). Relating the accidental perforations to the total number of 172 sinus augmentation surgeries, the incidence was 26% (corresponding to 45 unintentional perforations).

Two techniques were used to seal the maxillary sinus mucosa, namely, collagen membrane coverage and suturing of the Schneiderian membrane.

In the group of subjects with a perforated or incised Schneiderian membrane, suturing was performed for 31 patients (51%), and collagen membranes were used for 30 patients (49%). Among the patients who underwent suturing of the sinus mucosa, five (16%) had failed sinus grafts; two of these five failures were caused by incorrect design and support of the provisional restoration, which applied unfavorable pressure to the surgical site. Among the surgeries involving Schneiderian membrane suture, 26 cases (84%) were shown to be a success.

For Schneiderian membrane sealing by collagen membrane coverage, successful sinus graft integration was achieved in 28 cases (93%), while two cases (7%) resulted in failed sinus graft consolidation (Table 3). A Fisher’s exact test shows that there was no statistical significance (*p* = 0.425) regarding the success rate of both techniques.

In only six out of the 61 Schneiderian membrane perforation cases, implants were immediately inserted. The other 55 were planned as two-stage cases: sinus floor augmentation, followed by implant insertion after 5 months. The implants used for these patients varied in brand, length and diameter, and included: Zimmer Biomet (3.75 mm and 4.1 mm in diameter and 13 mm in length), Dentium (3.6 mm and 4.1 mm in diameter, and 14 mm in length); Straumann (3.3 mm and 4.1 mm in diameter and 14 mm in length); and ARDS (3.75 mm and 4.2 mm in diameter and 13 mm in length).

For both techniques, implant survival was first assessed one year after prosthetic restoration. The patients were followed up every 6 months for 1 to 7 years, with a mean follow-up period of 3.5 years.

Patients with successful sinus graft presented good healing after each surgery. Clinically evaluated during the first month after surgery, the soft tissue in the maxillary sinus area had a normal color and texture, and presented good healing with no clinical sings of infection, and no fistula or exposed xenograft granules. During the first 7 days, patients described no immediate pain or minimally tolerable pain post-surgery.

Reentry for implant insertion, after a minimum of 5 months, revealed good healing both of the soft tissue and the grafted site. The surgical site presented normally colored gingiva, no signs of inflammation, no fistula and no bone exposure. The bone underneath the soft tissue had a normal color as well, bled after drilling and allowed good primary stability for the implants.

CBCT examinations were performed 5 months after the sinus floor augmentation showed an uniform distribution of the graft material, well-delimited by the bone plates and the Schneiderian membrane. The CBCT examinations revealed no bovine bone granules beyond the sinus membrane and no sign of maxillary sinusitis.

Uncovering the implants revealed good integration, no mobility and no marginal bone loss. Further on, prosthetic success was achieved through correct implant placement and alignment in the grafted site.

## 4. Discussion

Perforation of the Schneiderian membrane is a common complication of sinus floor augmentation, with an incidence of 6.0–10.9% for bone scrapers and ultrasonic devices. Among them, the erosion technique is the safest, with an incidence of 4.7% [4]. Studies with bone window outlining and its reflection show a perforation incidence of 17.6% in comparison with rotary instruments [4]. When the bone window is removed, perforations occur in 9.6% cases [4]. In this study, the incidence for accidental perforation among the entire group of sinus augmentation surgeries was very high (26%) when compared with piezoelectric antrostomy. For many accidental perforations, tearing occurred during elevation of the membrane, due to sinus septa or to a very thin Schneiderian membrane, and not at the point when the antrostomy was performed. Current surgical sealing techniques, other than suturing, include the use of demineralized laminar bone, oxidized cellulose, biologic glues, PRF membranes or absorbable membranes (collagen membranes) [3,14,15].

Both maintaining the integrity of the Schneiderian membrane and sealing off perforations are critical for the success of bone augmentation procedures in the maxillary sinus [16]. According to different studies, the survival of implants diminishes when they are inserted in augmented maxillary sinuses with perforated membranes [12,17,18,19].

Experimental studies have demonstrated the Schneiderian membrane to be a source of humoral factor intake and osteoprogenitor cells for the neoformation of bone tissue [20]. However, histological studies have shown an insignificant osteogenic influence of the sinus membrane when compared to surrounding bone [21,22]. Histological human studies have proven an inverse proportion between vital bone formation and the buccal-palatal distance [23], and, respectively, a larger sinus needs a prolonged time for maturation of the graft material [24,25,26].

Animal studies have shown that bone formation is not related to direct contact with the sinus membrane, but it begins on the bottom of the sinus cavity after the elevation of the Schneiderian membrane and spreads into the elevated space [27]. Studies on monkeys offer insight into a major role in new bone formation to the coagulum, which occupies the space under the elevated Schneiderian membrane [28]. The sinus membrane does not provide a basis for new bone formation [28].

Vascular anastomosis by the union of the infraorbital artery branches and the posterior superior alveolar artery contribute to the vascularization of the Schneiderian membrane and, especially, of the lateral wall of the maxillary sinus [29]. Creating an access window to the maxillary sinus consequently reduces the surface of the receiving bed. Therefore, the sinus membrane is crucial for the blood supply corresponding to neoangiogenesis at the level of the bone-grafting material [16].

Tearing of the membrane frequently occurs near the bony contour of the antrostomy window. This is a consequence of inappropriate removal of the bony fragment or direct injury to the membrane by the insertion of the ultrasound bone surgery insert. The ultrasonic device provides high precision without traumatizing soft tissue, and reduces postoperative discomfort. Bone cutting was performed under constant irrigation using a cooled saline solution. The insert used has a micro-saw shape that leaves behind small grooves and thus allows delimitation of the bony window, which might be used for simultaneous lateral or vertical ridge augmentation.

The inserts cut through the bone by linear, continuous movements, under their own weight, without applying any additional pressure. Although ultrasound surgery devices should not harm soft tissues, the light pressure of the micro-saw insert can produce microscopic perforations in the underlying membrane that will allow it to tear even at minimum stresses during elevation by hand with specialized instruments [30]. Similarly, the sharp edges of the bony window can damage the sinus membrane in the same way, reducing its resistance during elevation [31].

Likewise, retractors used to reflect the surrounding tissues located at the upper edge of the access window may cause accidental iatrogenic perforations. Langenbeck and Farabeuf retractors are commonly used for mucoperiosteal flap retraction during oral surgery. The Langenbeck retractor ensures visibility at the surgery site by mechanical traction and reduces hemorrhages by putting pressure on incised tissues [32]. However, incorrect use of these retractors may cause complications such as mucosal perforations, resulting in additional technical difficulties and prolonged surgical intervention. In Figure 3, an iatrogenic sinus membrane perforation is exemplified. Because of muscle fatigue, the assisting physician who retracted the flap to ensure visibility slipped the Langenbeck retractor into the sinus cavity. As a result, membrane tearing occurred at the upper edge of the bone contoured similar to the size of the retractor.

No patient from the entire group had allergies to amoxicillin. On the other hand, the use of clindamycin and chlorhexidine was avoided, because some studies (Khoury F. et al.) consider clindamycin to be a risk factor for infections following sinus floor augmentation, while some suggest that chlorhexidine interferes with graft material consolidation [33].

Independent of the cause of Schneiderian membrane perforations, both surgical sealing techniques presented high survival rates of the implants. The chosen treatment technique was determined by the size and location of the sinus membrane rupture. The location of the perforation along the lateral window was important in terms of surgical access for sealing. The size of the membrane perforations was important because of the distance between the membrane margins to be rejoined; the larger the membrane rupture, the greater the risk of producing additional perforations.

For large-sized and multiple perforations, the pericardium membrane was chosen, while medium- and small-sized perforations were sutured. In addition, perforations located superiorly next to the maxillary sinus roof allowed suturing of the membrane, while inferiorly positioned ruptures were prone to collagen membrane coverage (Table 4).

Large (more than 10 mm) or multiple perforations usually occur in thin, fragile sinus membranes with reduced elasticity. The remaining mucosal tissue between multiple perforations makes it impossible to suture without additional strain on the membrane. In addition, medially positioned perforations from the osteotomy contour lead to reduced access and visibility, which hindered correct suturing of the mucosal margins. For these reasons, the treatment of choice in this type of Schneiderian membrane tear is to seal with a pericardium membrane in order to provide an inside lining (Figure 1).

The suturing technique is delicate, and requires first that the membrane be very well detached from the bone and relaxed so that it can be tension-free after suturing. A number of special instruments are needed: magnification with lights, microsurgical needle holders for 6.0–8.0 sutures, two microsurgical diamond-dusted tissue pliers and microsurgical scissors. Because the membrane is positioned behind the bony window, it is usually quite difficult to use fingers for knot-tightening without exerting tension at the membrane level. It is better to use diamond pliers to create the knots. When the perforation is solely in the membrane, a mattress suture is used. First, the needle (which is held by the microsurgical needle holder) enters the lower part of the perforation, and then is caught inside the sinus with the diamond pliers. The needle is again fixated into the needle holder and perforates the upper segment of the membrane. It is not advisable to pass the needle directly through both membrane segments at once, because this action usually creates tension and enlarges the perforation. After the needle is again fixated in the needle holder, the same operation is again carried out to obtain a mattress suture. Then, both ends of the thread are caught with both diamond pliers, and all three knots are fixed with them.

When suturing the membrane to the upper bony edge, first the needle is pushed through the hole made in the bone, then retrieved from inside the sinus cavity. After the needle perforates the membrane from inside the sinus cavity, it is removed from the membrane and pushed back inside the sinus. This is the most difficult part, when the needle has to emerge from the sinus through the small hole. By doing so, a mattress suture connects the membrane firmly to the bone without creating any tension at the membrane.

Other authors have proposed collagen membrane coverage for defects smaller than 5 mm and additional sutures for larger defects [34]. Testori et al. (2008) described the absorbable collagen membrane as a viable option to obliterate large perforations, creating a new superior wall or a “pouch” that can contain the entire number of bone grains [35].

Another technique for relatively small perforations includes fast absorbable collagen membranes, or simply leaving the Schneiderian membrane to overlap itself [17]. A study performed in 2015 examined 99 implants inserted in the augmented maxillary sinus by a lateral approach, with Schneiderian membrane perforation and collagen membrane coverage [36]. The mean follow-up duration after implant placement was 2.2 years, and the results proved a cumulative implant survival rate of 100% [36].

PRF membranes are considered an alternative treatment option for collagen membrane coverage [15]. Studies have shown no significant differences in the histological analyses of a healed Schneiderian membrane perforation with PRF or collagen membranes [15]. Suturing is the best option in terms of the osteogenic process, allowing direct contact between the graft material and the sinus membrane. The use of pericardium membrane increases both the osseointegration time of the graft material and the costs of the surgery, while suturing prolongs the surgical procedure but reduces the time required for osseointegration of the graft material.

In animal studies (rabbits), bone graft consolidation was analyzed through microcomputed tomographies and histomorphometries. The results showed delayed bone formation for sinus grafts with Schneiderian membrane perforation and collagen membrane sealing in comparison with an intact sinus membrane [37].

Quality of bone graft consolidation is associated with the amount of vital bone formed in the scaffold of the grafting materials [38]. In sinus floor augmentation, success is more related to a sufficient blood supply of the augmentation site and to an intact periosteum, with its anatomical and physiological properties, than to any specific grafting material [38]. Suturing the sinus membrane is a good alternative in terms of the osteogenic process, allowing direct contact between the graft material and the Schneiderian membrane. The causes of failure for this technique were insufficient blood supply, incorrect design of the provisional restoration, low healing potential after previous infections and inefficient sealing of the membrane perforations.

Patients with failed sinus grafts presented suppuration and signs of inflammation in the third or fourth week after surgery. In those cases, the bone graft material was removed, followed by a period of 6 months healing. The sinus floor augmentation surgery was repeated, usually with simultaneous implant placement. Only in two cases where the residual height bone was less than 4 mm did we postpone the implant placement. At the end, all patients received implant-supported fixed prosthesis.

All sinus floor augmentation surgeries without Schneiderian membrane perforation (111 sinus floor augmentation, corresponding to 65% from the total number of 172 sinus floor augmentation surgeries) were performed as planned, without intra- or postoperative incidents. Implants were inserted simultaneously with the sinus floor augmentation (when residual bone height allowed a good primary stability), or delayed until 5 months after the surgery. After the implants were integrated, fixed, screw-retained restorations were performed.

Within the limitations of this retrospective study, we can conclude that suturing the Schneiderian membrane might be considered a skillful alternative compared to other techniques with an external barrier applied between the augmented site and the graft material.

## Figures and Tables

**Table 1:** Study group characteristics.

Inclusion Criteria	Exclusion Criteria
Sinus floor augmentation by a lateral approach (height of residual bone less than 5 mm in posterior area)	Implant placement performed subsequently in other clinics
Schneiderian membrane perforation during the procedure	Prosthetic restoration performed in other clinics
Preoperative and postoperative CBCT (cone beam computed tomography) (6 months after surgery)	Patients who refused to be included in the study

**Table 2:** Distribution of the perforations among the sinus floor augmentation surgeries.

Total Surgeries	172
Perforations	61 (35%)
Unintentional perforation	45 (74%)
Intentional membrane incision	16 (26%)

**Table 3:** Results of the two sealing techniques.

Schneiderian Membrane Sealing Technique		Failure	Success	Bone Density at 5 Months (Cutting Resistance)
Repair by suturing	31 (51%)	5 (16%)	26 (84%)	D3
Repair by membrane sealing	30 (49%)	2 (7%)	28 (93%)	D4

**Table 4:** Sealing techniques depending on the size and location of the perforation.

Sealing Technique	Type of Perforation	Position in the Bony Window Osteotomy	Number
Collagen membrane	large (>10 mm)	center; inferior	8
	medium (5–10 mm)	inferior	22
Suture	medium (5–10 mm)	center, superior	16
	small (<5 mm)	center, superior	15
